# Nurse-patient communication in primary care diabetes management: an exploratory study

**DOI:** 10.1186/1472-6955-12-20

**Published:** 2013-09-13

**Authors:** Lindsay Macdonald, Maria Stubbe, Rachel Tester, Sue Vernall, Tony Dowell, Kevin Dew, Tim Kenealy, Nicolette Sheridan, Barbara Docherty, Lesley Gray, Debbie Raphael

**Affiliations:** 1 Department of Primary Health Care and General Practice, University of Otago, Wellington South 6242, New Zealand; 2 School of Social and Cultural Studies, Victoria University of Wellington, Wellington 6012, New Zealand; 3 University of Auckland, Auckland, New Zealand

## Abstract

**Background:**

Diabetes is a major health issue for individuals and for health services. There is a considerable literature on the management of diabetes and also on communication in primary care consultations. However, few studies combine these two topics and specifically in relation to nurse communication. This paper describes the nature of nurse-patient communication in diabetes management.

**Methods:**

Thirty-five primary health care consultations involving 18 patients and 10 nurses were video-recorded as part of a larger multi-site study tracking health care interactions between health professionals and patients who were newly diagnosed with Type 2 diabetes. Patients and nurses were interviewed separately at the end of the 6-month study period and asked to describe their experience of managing diabetes. The analysis used ethnography and interaction analysis.

In addition to analysis of the recorded consultations and interviews, the number of consultations for each patient and total time spent with nurses and other health professionals were quantified and compared.

**Results:**

This study showed that initial consultations with nurses often incorporated completion of extensive checklists, physical examination, referral to other health professionals and distribution of written material, and were typically longer than consultations with other health professionals. The consultations were driven more by the nurses’ clinical agenda than by what the patient already knew or wanted to know. Interactional analysis showed that protocols and checklists both help and hinder the communication process. This contradictory outcome was also evident at a health systems level: although organisational targets may have been met, the patient did not always feel that their priorities were attended to. Both nurses and patients reported a sense of being overwhelmed arising from the sheer volume of information exchanged along with a mismatch in expectations.

**Conclusions:**

Conscientious nursing work was evident but at times misdirected in terms of optimal use of time. The misalignment of patient expectations and clinical protocols highlights a common dilemma in clinical practice and raises questions about the best ways to balance the needs of individuals with the needs of a health system. Video- recording can be a powerful tool for reflection and peer review.

## Background

Diabetes is a major health issue for individuals and health services globally. In New Zealand diabetes is one of the most important conditions routinely seen in primary care settings, and disproportionately affects Maori and Pacific peoples, and those from low socioeconomic backgrounds [1-3]. There is a considerable literature internationally on the management of diabetes and also on communication in primary care consultations [4-6]. However, there are few studies which combine these two topics, whether generally or more specifically in relation to nurse-patient communication.

Optimal management of diabetes in primary care in New Zealand involves patients engaging with the combined and coordinated efforts of several health professionals (GP, nurse, podiatrist, dietician and retinal screening services), with referral to secondary services as needed [2,7]. In New Zealand, diabetes care is largely funded through CarePlus [8], a chronic care initiative introduced in 2004 as part of the New Zealand Primary Health Care Strategy [9]. This initiative provides additional funding for primary health organisations to give care for people with high needs because of chronic conditions or terminal illness. The specific aims are to improve management of chronic conditions, reduce inequalities, improve teamwork among health professionals and reduce cost of services for high need users [8].

Practice nurses have a vital role in the initial management of Type 2 diabetes in primary care, which is largely directed towards assisting patients to understand the nature and possible trajectory of the disease, and to become self-managing. The approach taken by practice nurses involves assessments, goal-setting, and information-sharing about self-management in more extended face to face consultations which inevitably turn to conversations concerning lifestyle, behaviour modification and risk reduction [10].

However, this approach is, as yet, unfamiliar to many patients who are more used to a problem-oriented medical system which deals with immediate rather than long-term concerns, even in the context of long-term conditions. A related problem arises from the complexity of diabetes and diabetes management. Previous studies have reported that misunderstandings and mismatches in expectations between patients and health professionals may arise due to the different priorities of disease management protocols in diabetes care and a patient centred consulting approach [11-14]. This is compounded by the fact that there may be very few symptoms at the time of initial diagnosis, or ones which are of gradual onset.

From a nursing perspective, an important part of diabetes care lies in eliciting and imparting information in a timely way through face-to-face consultation. Nursing consultations are supported by New Zealand guidelines for best practice in diabetes care, including various checklists [2] which have been widely disseminated and taken up by health practitioners. The guidelines are intended to inform primary care practitioners on targets of treatment and management and therefore go some way to support delivery of a comprehensive and consistent service.

It is easy for practitioners to regard the guidelines with their associated checklists as prescriptive of what *must* be accomplished in a given consultation or series of consultations, rather than starting from the patient’s existing knowledge and immediate concerns [15]. Many patients have difficulty assimilating the knowledge that diabetes can lead to significant adverse effects on many different parts of the body. Similarly, making changes to lifetime habits of diet and exercise is not easily accomplished and this is especially so when there is no immediate apparent need. As a consequence, close adherence to guidelines by means of a checklist or e-protocol may become unhelpful in individual consultations [15,16].

Making checklists the focus of a consultation is also at odds with how nurses prefer to relate to patients more generally. Everyday nursing work, including diabetes management, is mediated through talk [17], and there is increasing recognition in the research literature that nurse-patient encounters have both a content component and a relational component, both of which are important [18-20]. However, in spite of an increased awareness of the need for patient-centred approaches, research suggests that in practice such approaches do not occur as often as might be beneficial [20-24].

It is well-accepted that the quality of communication between health practitioners and patients can make a significant difference to health outcomes [20,25]. There is also good evidence that practitioners can improve the quality of care they provide by better understanding the consultation process and focussing on effective communication both within consultations and over time [20,26-29].

In teasing out exactly how communication affects health outcomes, Street et al. [20] describe what they call the “proximal outcomes of the interaction (e.g., satisfaction with care, motivation to adhere, trust in the clinician and system…) that could then affect health or that could contribute to the intermediate outcomes (e.g., adherence, self-management skills, social support) that lead to better health.”p.297.

The study reported here set out to examine the actual talk and perspectives of nurses and patients who were newly-diagnosed with diabetes in order to describe the features of effective interaction and to identify areas for reflection and possible improvements to practice. Criteria for effective communication included both: (1) video evidence of smooth comfortable interaction at an interpersonal level within the consultation, as demonstrated by verbal and non-verbal cues and (2) recorded evidence over the 6 month period of some retention and appropriate recall of information exchanged.

## Methods

### Study design

This paper reports on an analysis of 35 video-recorded consultations between 10 nurses and 18 patients, and associated interviews. These consultations and interviews are part of a larger parent study concerning communication with diabetes patients [30]. The larger study entitled “Understanding Diabetes Management: Tracking Communication in Primary Care” was given ethical approval from the Lower South Regional Ethics Committee of the New Zealand Ministry of Health Reference Number LRS/08/09/041.

### The parent study

The parent study tracked and recorded all health consultations (N = 116) and associated communications relating to 34 patients who were newly diagnosed with diabetes, over a period of six months. Data gathered included video and audio recordings of patient consultations with GP, nurse, dietician, podiatrist, health psychologist and eye screening services along with field observations, demographics, medical records and interviews with patients and health professionals at various stages of each six-month case. Interview data included short debriefs after each recorded consultation and full semi-structured exit interviews with key participants at the end of the six-month data collection period. Nurse and GP participants were interviewed about their experience of caring for people with diabetes; likewise, patients were interviewed about their experiences and expectations of managing diabetes. In addition, some nurse and GP participants viewed and commented on short clips of their own consultations as part of the development and piloting of a toolkit for effective diabetes communication.

A total of 62 nurse-patient consultations were recorded in the multi-site parent study. The 35 consultations reported on here are all those collected from one geographical arm of the multi-site study and are representative of the demographic diversity of the entire data set (see below).

One of the research team (LM) did most of the recording and ethnographic fieldwork for this arm of the wider study, and was, as a consequence, very familiar with the context and nature of the data.

### Recruitment and data collection

General medical practices which had already shown an interest in research or were likely to have a high number of patients with diabetes were invited to participate. Practices were selected to ensure a demographically diverse population base. In consultation with the practice, and with their agreement, researchers approached potential patient participants who had given verbal indication of interest to one of their primary care practice team. The researcher supplied written information, explained the study in detail, and gained written consent from each participant prior to any recording.

Eligible patients were adults who had a new diagnosis of Type 2 diabetes and were able to give informed consent. All participants were in the study for a period of 6 months following a new diagnosis of Type 2 diabetes, but this time period did not always include the initial GP consultation where the diagnosis was first discussed and confirmed following a blood test. In several cases it was apparent that patients had been in a ‘pre-diabetic’ state for up to two years earlier, as measured by a rising trend in blood HbA1c.

Nurses in participating practices were recruited in two ways. They either initiated contact with the researcher themselves or became drawn in to the research because an existing patient participant was scheduled to have a consultation with them at some point in the 6 month period of data collection.

Ten nurses and 18 patients participated in this geographical arm of the study. The sampling frame for the wider study aimed for diversity in terms of age and ethnicity. Patients in this arm included 10 females and 8 males with ages ranging from 32–89 years. The majority (N = 14) were in the 40–65 age group, with the remaining 4 patients in their 30s (N = 2), a 70 year old and an 89 year old. A wide range of ethnic groups was represented, including NZ European (N = 6), Maori^a^ (N = 3), NZ European/Maori (N = 1), Indian (N = 3), and one patient from other ethnic groups (Chinese, Samoan, Fijian, Assyrian and Eritrean).

Video and audio recording devices were placed in the consultation room for each participant consultation and removed at the conclusion of the consultation. This was a method used successfully in previous studies [31] and ensured data from each participant consultation could be obtained with minimal disruption to the work of the practice. Participants were reminded that they could stop the recording at any stage without having to give a reason. A researcher was not present in the consultation.

### Data analysis

Analysis followed an iterative qualitative process including content analysis, interactional analysis of consultations and interviews, and closer interactional and observational analysis of selected segments supported by field notes and associated medical records.

The unit of analysis for the study was the individual set of ‘episodes of care’ for each patient over the observation period. Analysis encompassed both micro-level analysis (fine-grained linguistic and sequential analysis of individual interactions and texts) and broader ethnographic descriptions of the institutional context of diabetes care and its communication patterns and systems.

Following data collection (LM), each recording was viewed and the content summarised in a detailed written log by an independent nurse researcher (SV). Research assistants transcribed the interactions. In addition, data for each patient was labelled, anonymised and collated into an inventory which included length of recordings for each interaction (RT). An overall picture of the cohort of patients was gained by plotting the number and length of consultations each patient had during the 6-month study period (LM and RT). This provided context for analysis of the nurse-patient consultations.

More in depth content and interactional analysis started with data sessions where a multidisciplinary team with backgrounds in general medical practice (TD, TK), nursing (LM, SV, NS, BD ), interactional sociolinguistics (MS), information management (RT), psychology (DR) public health (LG) and sociology (KD), viewed consultations and noted anything at all that stood out as unexpected, curious or interesting. Initially these sessions provided opportunity for closely observing the nature of routine practice and for open-ended, unconstrained enquiry.

Different analytic methodologies were used as follows. Interactional sociolinguistics was used to examine the fine-grained detail of the interactions at ‘key moments’ in the diabetic care pathway [32] to identify how communication cues lead to effective communication and/or misunderstanding. Document analysis and content analysis assisted in identifying particular communication themes and patterns that facilitate or hinder communication processes.This linguistic and ethnographic analysis was integrated with analysis of the interactions from a clinical perspective [33]. The aim was to develop a ‘thick description’ [34] of the way patients newly diagnosed with Type 2 diabetes negotiate the complexities of the NZ primary care system by triangulating data from different sources and using different analytic methods.

Phrases such as “a bit overwhelming” occurred several times in the recorded consultations and interviews and was said not only by patients but also by nurses. The research team remarked on how long some of the consultations were and on the large amount of information being offered both verbally and in written form. These initial impressions prompted more in depth data analysis of the context and possible explanations for participants expressing a sense of being overwhelmed. The data inventory was viewed and the logs, consultations and interviews were searched for the term ‘overwhelming’. Quantitative data was checked, e.g. lengths of consultations (RT) and each recording or interview where the “overwhelming” expression occurred was reviewed (LM). This iterative process involved closing in on small segments and then drawing back to explore contextual data to help understand the nature of communication in those instances.

## Results

The results are grouped into three main categories:

1. A brief description of the number and length of consultations per patient

2. Content of consultations and use of checklists or protocols

3. Participant expectations and perceptions

### A brief description of the number and length of consultations per patient

An overview of the contact each patient had with health professionals in the 6-month study period showed that patients spent more time with nurses than with any other health professional. Of the 18 patients in this study, and in the 6 month study period, 11 had one consultation with a nurse alone and 6 had 2 consultations with a nurse alone. There were 11 consultations with a nurse where the GP joined in for part of it. One patient had a consultation with nurse and dietician together (See Table 1).

**Table 1:** Types of nurse-patient consultation

Nurse alone	23
Nurse and GP	11
Nurse and Dietician:	1
**Total**	**35**

**Figure 1:**
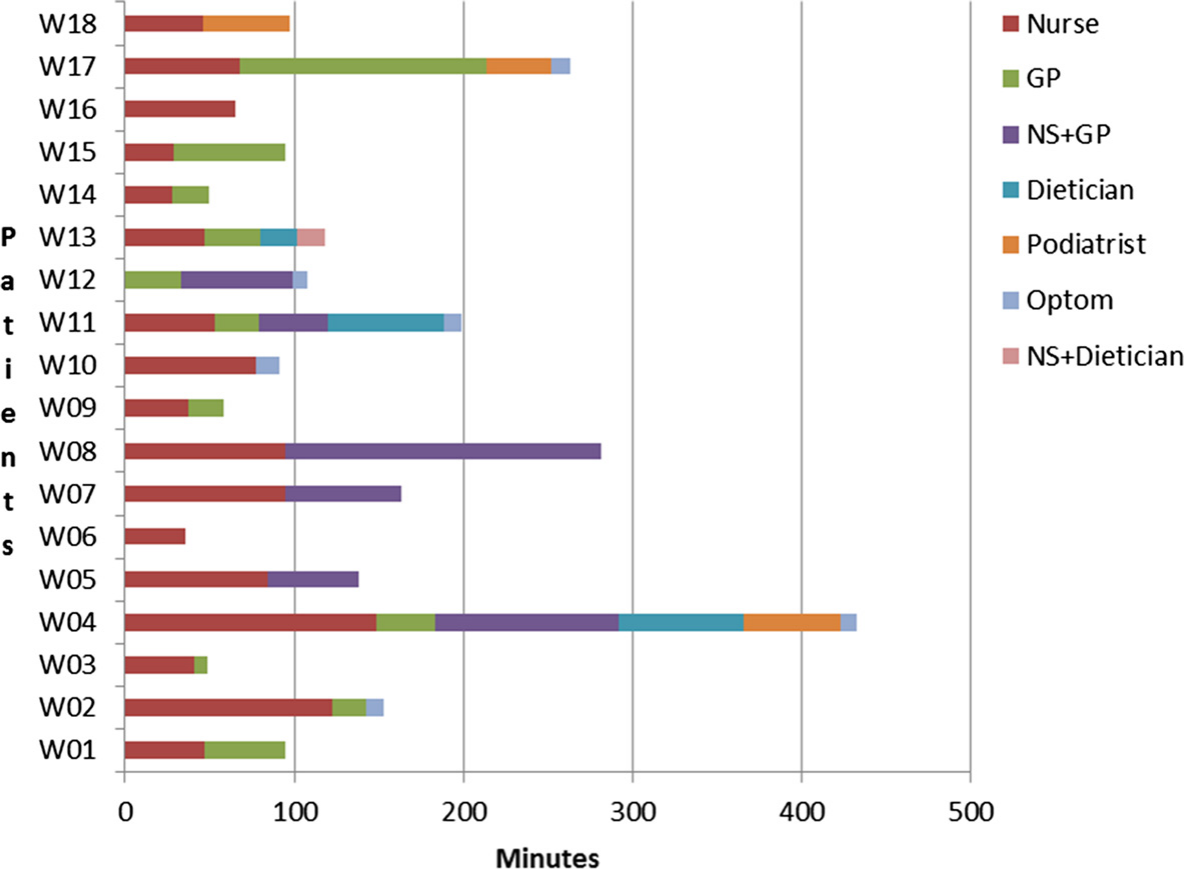
Individual profiles of recorded contact with health professionals over 6 months.

Individual consulting patterns of the 18 patient participants over the 6 month study period are represented in Figure 1 below. (Note that consultation lengths have been aggregated by health professional and do not reflect the order of consultation.) Initial consultations with nurses were longer than with other health professionals involved in diabetes management (average length 52 minutes, range 8–95 minutes) compared with an average 21 minutes with a GP, range 8–35 minutes.

These figures take into account only those interactions actually recorded - our study may not have captured all the interactions these patients had in the 6 month time frame. For 7 patients the initial consultation with their GP was not recorded.

**Figure 2:**
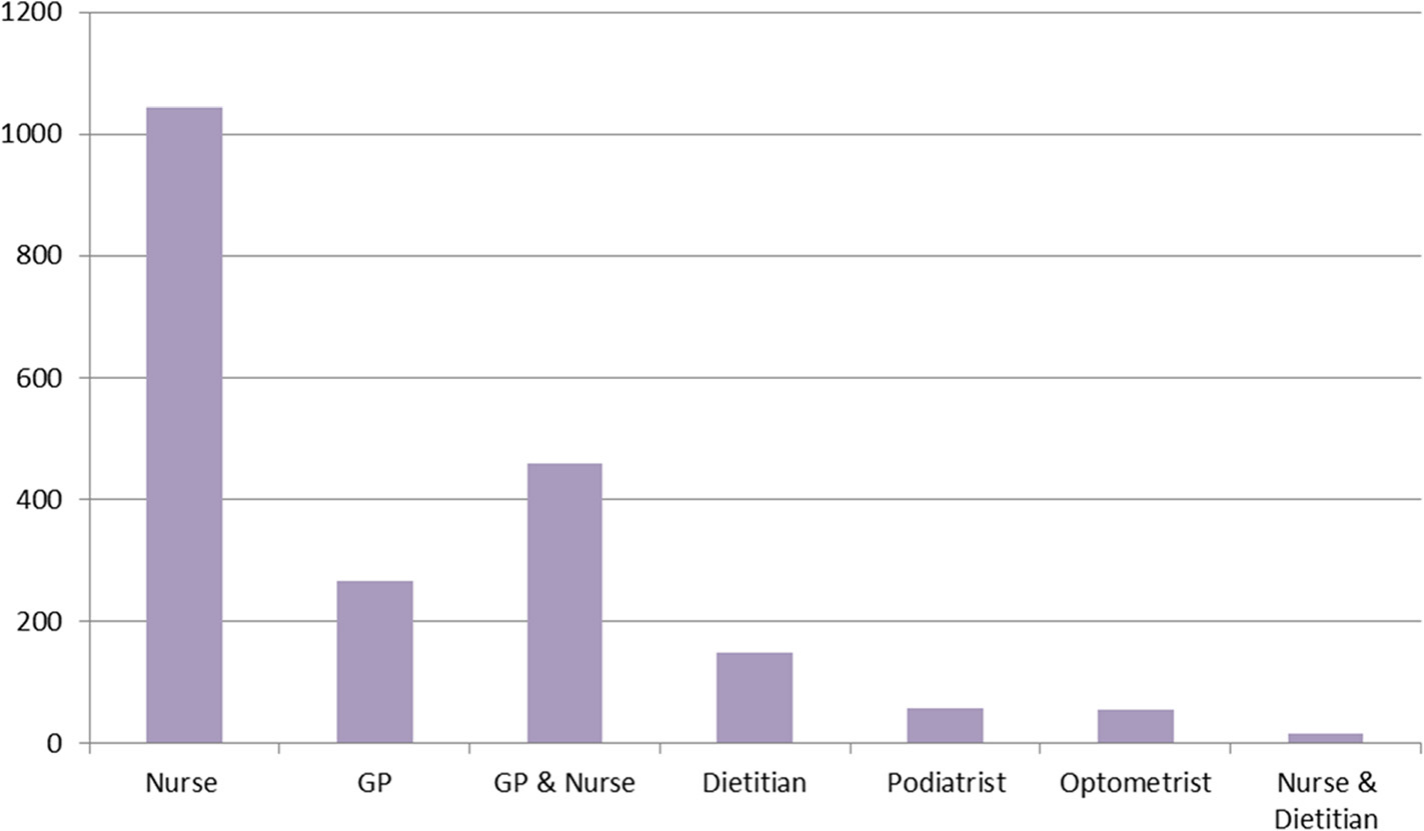
Total time, in minutes, spent with health professionals in the 6 month study period.

Figure 2 shows the aggregated total time that the 18 patients in this study spent in consultation with each health professional, counted by the minute. The time spent with nurses, either alone (over 1000 minutes in total) or together with another health professional (almost 500 minutes in total) was significantly higher than for any other health professional.

### Content of consultations and use of checklists and protocols

#### Content

The clinical content of nurse-patient consultations was wide-ranging as might be expected at the outset of a chronic illness such as diabetes. There are some physical examinations (e.g. sensation and skin integrity of the feet), and the imparting and eliciting of a large amount of information on physiology, risk, lifestyle and on-going care. A typical list of topics covered includes: diet, exercise, weight, eye-screening, depression, blood sugar monitoring, dental hygiene, cardiovascular risk, skin care and sexual health. As well as these physical topics, the data shows that nurses take care to address social issues and services and attend to interpersonal relational work.

Nurses, in the interview data, reported that they felt compelled to discuss and/or provide information on a large number of topics in one or two initial consultations because diabetes affects so many aspects of well-being and there may be only one opportunity with the patient. This is illustrated in the following typical excerpts taken from interviews. Both nurses quoted below describe the same dilemma and illustrate their conscientious attitude.

Excerpt 1*Nurse: I think the problem is we probably say too much…but then when you get someone like [name ] you know you’re probably only going to have one chance.*

Nurse interview. **DS-RS22-03c_NS11_Interview**

Excerpt 2*Nurse: They mightn’t come back for appointments; you might see them once.*

Interviewer: Does that happen much?

Nurse: You’re often chasing, yeah a lot, you’re chasing people up, they don’t see the need…that’s all part of primary health.

Nurse interview. **DS-RS17-12_NS16_Interview**

Nurses were well aware of the dilemma they faced in wanting to impart as much information as possible while remaining sensitive to what patients could deal with at a given time, as in the following examples.

Excerpt 3*Nurse: I like to cover cover everything and I know you can’t really cover everything.*

Nurse interview **DS-RS01-30_NS13_Interview**

Excerpt 4*Nurse: She seemed to take on board what I said and um yeah very hard to know how much to give her though and whether you give her the right stuff.*

Nurse interview **DS-RS22-03b_NS17_Interview**

The data strongly suggests that this large volume of information may be difficult for patients to take in initially. Patients expressed some surprise and bewilderment after initial consultations, as in the example below.

Excerpt 5*Patient: she [the nurse] wants me to have breakfast and stuff like that but it*

GP: mm

*Patient: you know i don’t really understand diabetes….* [2 lines omitted]

GP: mm mm yeah did [Nurse] go through that little book with the pictures with you about [diabetes]

Patient: er she showed me some but oh it was just quite overwhelming

GP-Patient consultation **DS-GP18-01**

This was a fairly common theme and words and phrases like ‘overwhelming’ ‘a bit of a lecture’, ‘talked at’ recurred throughout the interactions and interviews.

#### The use of checklists and protocols

On viewing each consultation and looking for the clinical content singly and across the 6 month time period for each patient, it quickly became apparent that most nurses explicitly referred to or had adopted and internalised the national guidelines [2,7] for diabetes management in some way. They generally used an electronic protocol or checklist, derived from the national guidelines, to structure the consultation.

Checklists helped nurses and patients by making sure essential clinical and well-being issues were addressed in a consistent way and by normalising or sensitively broaching delicate issues like mental health, as in this example, 43 minutes into the consultation:

Excerpt 6*Nurse: there are a few other areas which I’ll just go through as well one is ((tut)) ((inhales)) there has been a connection made between diabetes and depression ((PT nods)) ((inhales))((tut)) um ((inhales)) is um ((tut)) ((inhales)) do you have any issues with mood no*

Patient: no

Nurse-Patient consultation **DS-NS13-03**

(Note that the ((tut)) in Excerpt 6 signifies a non-specific verbal hesitancy like a click of the tongue. It is one interactional indicator that this is a delicate issue.)

The nurse began by framing depression as just one of a number of topics which would be routinely discussed. She stopped short of baldly naming depression then rephrased it. The use of the passive voice (*there has been a connection made*) distanced the nurse from suggesting that this patient had depression. In this way, the nurse skilfully left the topic open for the patient to take up. While there was no further discussion of mental health in this consultation, the patient himself raised it in a subsequent consultation with the same nurse.

However, a degree of interactional discomfort arising from the use of checklists was also observed in many consultations. They were observed to constrain interactional flow at times and to override immediate concerns and usual interactional processes or responsiveness. In the following example, the nurse and patient had been exploring the topic of depression in some depth, when the nurse brought the discussion to an end by moving on to a completely unrelated topic, teeth, which was the next item of the electronic protocol.

Excerpt 7*Nurse: so in the last month you wouldn’t say you’ve felt ((inhales)) s- you know sad or down or not looked to forward to things as much as you normally would ((inhales))*

Patient: ((inhales)) no not really um i mean i’ve had a pretty hectic um personal life and that recently but ((inhales))

Nurse: yeah

Patient: um + i i think i coped with that quite well i don’t seem to go up and d-

Nurse: yeah

Patient: particularly mainly i think cos i vocalise a lot

…… 33 lines omitted ……..

Patient: so no i i don’t think i do have tendency towards depression at all really

Nurse: okay that’s that’s good that’s good ((inhales)) um ((tut)) ((inhales)) teeth is the other area

Nurse-patient consultation **DS-NS13-03**

In the following example, as the nurse moved through a series of checks, the patient introduced a concern outside the usual scope of diabetes checks:

Excerpt 8*Patient: now is there any chance of…with the next blood test one should because of my age should have the um prostate blood test as well…*

Nurse: [starting to examine feet, no eye contact with PT] oh yeah well we can ask [doctor] if he can do that [matter of fact tone]

Patient: and also the mechanical…the physical test I’ve not had one either

Nurse: pulses are good [referring to feet]…ok so you’ll probably have to see [doctor] for that

Nurse-patient consultation **DS-NS16-01b**

The communicative strategy used by the nurse in the example above might be interpreted in a number of ways. One interpretation is that the nurse proceeds with her checklist paying minimal attention to the different concern raised by the patient and that the patient might find that reaction disconcerting. Another more positive interpretation is that the physical touch and averted gaze provided by the nurse gave the patient a very welcome opportunity to raise a delicate issue and her matter-of-fact tone normalised the issue. In this recorded interaction the nurse in fact returned to the concern later in the consultation and noted it for follow up.

These brief examples from the interactional analysis of the consultations in our sample illustrate how the use of checklists and protocols could both help and hinder the flow of nurse-patient consultations. There was also evidence that nurses in this study were aware of the dilemma that these posed namely that while they facilitated coverage of all key topics, they could also make the interaction less natural. This point is exemplified by the following excerpt taken from an interview with the nurse after she had viewed clips of her recorded consultations.

Excerpt 9*Nurse: When I watched the two clips the other day I thought there were some things that I would’ve done differently ah hmm mm not sure how but I was very aware of the fact..... that I have an agenda that I’m working through so I’m kind of half listening, half typing, half thinking about what I need to ask next to complete my list.*

Nurse interview **DS-RS01-30_NS13_Interview**

Our data suggests that when checklists and protocols are used more flexibly or with discretion, nurses are freed to practice in a more autonomous manner that is contingent on and sensitive to the local context of the discussion. For example, in the following excerpt the nurse explained in her interview that she abbreviated her planned discussion when she noted the patient losing concentration. As the nurse drew the consultation to a close, she provided the patient with an opportunity to respond to the information she had been sharing and/or to raise a new concern. This appeared to be designed to enable the nurse to assess how the patient was reacting to the information and how she might direct her efforts in future for this patient. When the patient simply acknowledged her summary, she prompted for a further response.

Excerpt 10*Nurse: so er it is important that we um ((tut)) that we monitor all of those things with you*

Patient: mm yep

Nurse: hmm things to think about it is a lot isn’t it

Patient: things you don’t want to think about

Nurse-patient consultation **DS-NS10-01**

### Participant expectations

Nurses were clear about the purpose of their consultations in prospectively managing long-term conditions such as diabetes and were equally clear that they generally adopted a checklist-driven approach. The main objectives, from the nurses’ point of view, as expressed in interviews and recorded in field notes, were to help patients avoid or reduce risk associated with diabetes and to assist patients to become self-managing. However, interactional analysis of the video-recorded consultations revealed that these objectives were rarely signalled or stated explicitly, nor was the patient’s expectation sought explicitly at the start of these consultations. There was evidence from field notes and interviews that there is still some way to go to match expectations between parties in nurse-patient consultations (see examples below).

Additionally patients have different needs in terms of pace and amount of information and the data showed that this was not always easy to assess at the outset, and may not be easily accommodated in a checklist-driven consultation. In the following example the patient was describing a desire for a gradual and methodical 10-step process:

Excerpt 11*Patient: I don’t really want to know what the end result is at step 2.*

Patient interview **DS-RS17-11_W07_Interview**

In the following excerpt a patient expressed frustration at her inability to participate in the consultation as much as she would have wanted, and at a lack of acknowledgement of her prior knowledge and actions.

Excerpt 12*Patient: I bristled a bit, I felt like I was being talked at and I wasn’t being asked …[what I knew about diabetes]…or even my first thoughts about it……*

I think the big thing is is about asking people where they’re at; I don’t think you should assume that people have done nothing.

Patient interview **DS-RS01-29_W12_Interview**

Nurses reported sensing these mismatches but also commented that knowing how to change their approach was a challenge, as evidenced in the following examples taken from a nurse interview.

Excerpt 13*Nurse: it’s all very well having a set standard thing we’ve got to follow but you’ve got to look outside of that [be]cause everyone’s got their own needs.*

Nurse Interview **DS-RS17-12_NS16_Interview**

Excerpt 14*Nurse: My most valuable lesson always is that I should listen more…listen to where they’re at and take it from where that point is.*

Nurse interview **DS-RS17-12-NS16_Interview**

However, it was apparent in many consultations that this stated awareness did not lead to a shift away from the checklist to engaging in a conversation that listened to the patient and focused on their most important concerns.

Another important observation was that patient perceptions can change over time. One of the strengths of the study design was the tracking over a 6 month time period which provided an opportunity to ask patients to reflect back on their initial consultation. In the following example, a patient recognised benefits which were not obvious to him at the outset:

Excerpt 15*Patient: You don’t know what to ask so it’s good that they will offer information about it that’s what you really do need.*

Patient interview. **DS-RS01-10_W01_Interview**

With hindsight, this patient saw value in what was initially ‘a bit overwhelming’. Nevertheless, the data shows that there is clearly scope for nurses to share information with patients in ways that take more account of individual differences.

## Discussion

Nurses play a significant role in the team of health professionals who manage patients with diabetes in New Zealand.

This study has shown, notwithstanding the absence of some GP data, that initial consultations with nurses are longer than with other health professionals and, in many but not all cases, are driven more by the nurses’ clinical agenda than by what patients already know or want to know. Clinical content includes some physical assessments and discussion of physiology, risk, lifestyle and on-going care within the wider and particular context of each patient’s social circumstances. Analysis of the consultations, particularly of the clinical content both singly and across the 6 month time period for each patient, shows that nurses generally use a checklist or protocol to structure the consultation. Our analysis showed that while nursing activity is demonstrably aimed at supporting patients to become self-managing, these checklists and protocols for diabetes management can both help and hinder the overall flow and effectiveness of the consultations. The scope and amount of information imparted by nurses was clearly identified as problematic by patients and by nurses themselves as reflected in phrases like “a bit overwhelming” used by both patients and nurses when talking about managing a new diagnosis of diabetes.

On the other hand, some nurses in the study seemed to have the checklist ‘in their heads’ and oriented their questions toward it while remaining flexible and responsive to the issues and mood of the patient. These were skilled and experienced nurses but, without reproducing the entire transcript of their consultation, it is difficult to illustrate their expertise. This observation suggests that while many nurses in the study were experienced in practical aspects of diabetes management, they were yet to develop the same degree of comfort and familiarity with conducting consultations.

Perversely, the very conscientiousness of nursing activity observed in this study, along with the funded health initiative ‘CarePlus’ [8] which provides nurses with protected time, free to the patient, may actually be counterproductive. And this is especially so when a checklist driven approach is adopted in the consultation as it can unintentionally foster a situation where a great deal of information can be imparted – thus overwhelming the patient. It is possible that this funding for patients to receive generous access to nursing attention is actually in place ahead of sufficient widespread professional development to support its best possible use. Reporting on a UK study of practice nurses and the facilitation of self-management in primary care, W. Macdonald [35] notes that nurses ‘seemed to lack resources beyond personal experience and intuitive ways’ p.191 and that, ‘in the absence of specific education and skills in chronic disease management, nurses tended to rely on their own idiosyncratic methods’ ( p. 195). Of course idiosyncratic methods can also be highly effective, and many nurses typically already possess an impressive amount of experience and skill as primary health care practitioners. Nevertheless while most nurses in this study had undertaken extensive on-going education in chronic disease management, many expressed a desire for additional professional development in the area of consultation skills and, indeed, many were motivated to participate in the study for this reason, seeing their participation as a starting point for reflective learning.

Checklists and protocols featured prominently in this study. This is not surprising given the part they play in the growing emphasis on evidence-based clinical practice. Empirical evidence of how checklists and protocols are employed in practice, as captured in the data for this study, is valuable in exposing both their positive and negative immediate and short-term effects.

Guidelines with standardised checklists are intended as a guide to best practice and therefore have many positive attributes. However, this study suggests that strict or formulaic adherence to such checklists can reduce flexibility and fail to allow for the unique demands of each health interaction. Interactional processes may therefore be less than optimal, and there is a possibility that professional-patient relationships and long term health goals may be compromised.

This small exploratory study has provided a number of valuable qualitative insights into the nature of interactions between nurses and patients, along with a snapshot of the contribution nurses make overall to the primary care team’s engagement with patients with diabetes. One limitation is that the patterns observed in length and type of consultation could not be statistically verified, as we were not able to record every consultation for each patient. In particular, for 7 of the 18 patients not all initial consultations with the GP were recorded; this means that time spent with the GP time has been conservatively counted. Despite this, the overall trend of shorter GP consultations and longer nursing consultations was clearly evident.

## Conclusions

The sense of being overwhelmed, reported by both nurses and patients in this study, appears to arise from the sheer volume of information exchanged, along with a possible mismatch between expectations of patient and nurse about the scope and purpose of the consultation. This highlights a common dilemma in clinical practice, namely, the potential for individual patient concerns and the priorities of a health service provider to be different. It raises questions about how the needs of individuals and the needs of a health system can best be managed, and the degree to which checklists can be used flexibly. In this study conscientious and skilful nursing work is evident, but at times the nurses’ efforts are possibly misdirected in terms of optimal timing or targeting. The findings from this study provide an opportunity for practitioners to reflect on their everyday work, to allow them to communicate more effectively with patients and develop ways in which chronic care management might be approached differently.

## Endnote

^a^Maori are the indigenous people of New Zealand, numbering approximately 16% of the total population.

## Competing interests

The authors declare that they have no competing interests.

## Authors’ contributions

MS, TD, KD, LM, TK and NS designed the study. LM, NS, BD and RT undertook fieldwork and data collection. SV viewed and reviewed recorded data and made written summaries of each item. All authors contributed to data analysis. LM and MS drafted the manuscript. All authors read and approved the final manuscript.

## Pre-publication history

The pre-publication history for this paper can be accessed here:

http://www.biomedcentral.com/1472-6955/12/20/prepub
